# Draft Genome Sequences of *Acinetobacter* and *Bacillus* Strains Isolated from Spacecraft-Associated Surfaces

**DOI:** 10.1128/genomeA.01554-17

**Published:** 2018-02-08

**Authors:** Arman Seuylemezian, Parag Vaishampayan, Kerry Cooper, Kasthuri Venkateswaran

**Affiliations:** a Biotechnology and Planetary Protection Group, California Institute of Technology, Jet Propulsion Laboratory, Pasadena, California, USA; b College of Agriculture and Life Sciences, School of Animal and Comparative Biomedical Sciences, The University of Arizona, Tucson, Arizona, USA

## Abstract

We report here the draft genome sequences of four strains isolated from spacecraft-associated surfaces exhibiting increased resistance to stressors such as UV radiation and exposure to H_2_O_2_. The draft genomes of strains 1P01SC^T^, FO-92^T^, 50v1, and 2P01AA had sizes of 5,500,894 bp, 4,699,376 bp, 3,174,402 bp, and 4,328,804 bp, respectively.

## GENOME ANNOUNCEMENT

*Bacillus horneckiae* strain 1P01SC^T^ was isolated from a spacecraft assembly clean room at Kennedy Space Center (KSC), where the Phoenix spacecraft was assembled (1). As previously reported, spores of this strain were resistant to UV_254_ radiation up to 1,000 J m^−2^. *Bacillus nealsonii* FO-92^T^ was isolated from fall-out particles collected from a spacecraft assembly facility at the Jet Propulsion Laboratory (2). Spores of FO-92^T^ have exhibited resistances to UV_254_ up to 300 J m^−2^, and vegetative cells and spores of this organism were resistant in up to 5% liquid H_2_O_2_ (2). *Acinetobacter radioresistens* 50v1 was isolated from the surface of the Mars Odyssey orbiter (3). Vegetative cells of this organism were capable of surviving a combination of stressors, including desiccation, up to 1,000 J of UV_254_ radiation, and up to 0.33 mg/ml of H_2_O_2_ (3). *Acinetobacter proteolyticus* strain 2P01AA was isolated from the Payload Hazardous Servicing Facility at KSC during the assembly of the Phoenix spacecraft (4). As reported previously, strain 2P01AA exhibited increased resistance to H_2_O_2 _exposure and survival in up to 320 mM H_2_O_2_ (5). Here, we report the first draft genome sequences of *B. horneckiae* type strain 1P01SC, *B. nealsonii* type strain FO-92, and two *Acinetobacter* species strains, 50v1, and 2P01AA, isolated from spacecraft hardware and associated surfaces.

Strains 1P01SC^T^, FO-92^T^, 50v1, and 2P01AA, were sequenced using a shotgun sequencing approach on the Illumina HiSeq paired-end platform. The reads were *de novo* assembled using CLC Genomics Workbench version 10.1.1, resulting in total genome sizes of 5,500,894 bp, 4,699,376 bp, 3,174,402 bp, and 4,328,804 bp, respectively. Genome statistics are given in Table 1 for all the strains. Annotations were produced using both the Rapid Annotations using Subsystems Technology server (6) and the NCBI Prokaryotic Genome Annotation Pipeline (7, 8) and visualized using the SEED viewer (9).

**TABLE 1:** Genome statistics of four microbial strains isolated from spacecraft hardware and associated surfaces

Strain	GenBank accession no.	No. of contigs	Genome size (bp)	*N*_50_ size (bp)	Largest contig size (bp)	GC content (%)	No. of rRNAs	No. of protein- coding genes	Coverage (×)	No. of filtered reads
*B. horneckiae* 1P01SC^T^	PISD00000000	104	5,500,894	93,836	456,652	37.48	1 (16S), 1 (23S)	5,708	326	11,967,132
*B. nealsonii* FO-92^T^	PISE00000000	92	4,699,376	104,758	307,322	34.67	6 (5S), 2 (16S), 3 (23S)	4,712	563	17,642,747
*A. radioresistens* 50v1	PISK00000000	125	3,174,402	65,829	186,990	41.59	4 (16S), 3 (23S)	2,930	428	9,077,106
*A. proteolyticus* 2P01AA	PISJ00000000	36	4,328,804	316,343	447,298	41.10	1 (5S), 1 (16S), 1 (23S)	4,040	575	16,609,733

The *Bacillus* strains 1P01SC^T^ and FO-92^T^ had 103 and 99 putative genes coding for dormancy and sporulation, respectively. Both strains had MutS, RecA, MutL, excinuclease ABC, beta-lactamase, and genes coding for the formation of persister cells (10). Strain FO-92^T^ had a prophage-associated DNA repair protein (RecT), six genes associated with spore DNA protection, exodeoxyribonuclease III, and a peroxide stress regulator (PerR). Strain 1P01SC^T^ had cold shock proteins (CspD and CspA) and a heat-inducible transcriptional repressor (HrcA).

*Acinetobacter* strains 50v1 and 2P01AA possessed putative genes coding for persister cell formation, heat shock and cold shock responses, superoxide dismutase, rubredoxin-NAD(+) reductase, and cobalt, zinc, cadmium, and arsenic resistance (11). Strain 2P01AA had putative genes coding for heme oxygenase (HemO) and four genes coding for quorum-sensing molecules, which initiate biofilm biosynthesis and adhesion (12). Strain 50v1 had genes associated with betaine and choline uptake, which further allow for increased water retention in the cells (13), as well as alkyl hydroperoxide reductase subunit C and a DNA-binding protein (Dps), which has been shown to protect organisms from oxidative stress (14).

### Accession number(s).

The genome sequences of all four isolates have been deposited at DDBL/EMBL/GenBank under the accession numbers listed in Table 1.
